# Effect of S-region mutations on HBsAg in HBsAg-negative HBV-infected patients

**DOI:** 10.1186/s12985-024-02366-2

**Published:** 2024-04-23

**Authors:** Hui Liu, Shuxiang Chen, Xin Liu, Jinli Lou

**Affiliations:** Clinical Laboratory Center, Beijing Youan Hospital, Capital Medical University, 100069 Beijing, China

**Keywords:** Hepatitis B Virus, Occult HBV infection, HBV S region, Mutation

## Abstract

**Background:**

Occult HBV infection (OBI) is a special form of hepatitis B virus (HBV) infection that may cause Liver cirrhosis and hepatocellular carcinoma, causing significant harm to patients. Given the insidious nature of OBI, it is usually not easy to be detected. Most of the samples currently studied are concentrated on blood donors, however, patients in this special state have not been fully studied. This project aimed to study the effect of HBV S region mutations on HBsAg in patients with clinical OBI.

**Methods:**

Collect 107 HBsAg-/HBV DNA + blood samples from Beijing Youan Hospital, Capital Medical University from August 2022 to April 2023. Next, the successfully extracted and amplified HBV DNA S regions were sequenced. Construct mutant plasmids to verify the cell function of the high-frequency mutation sites and explore the possible molecular mechanism.

**Results:**

Sixty-eight HBsAg-negative samples were sequenced, revealing high-frequency amino acid substitution sites in the HBV S protein, including immune escape mutations (i.e., sY100C、sK122R、sI126T、sT131P、and sS114T) and TMD (Transmembrane domain) region substitutions (i.e., sT5A、sG10D、sF20S、and sS3N). We constructed a portion of the mutant plasmids and found that sT5A, sF20S, sG10D, sS3N, sI68T, and sI126T single point mutations or combined mutations may decrease HBsAg expression or change the antigenicity of HBsAg leading to detection failure.

**Conclusions:**

HBsAg-negative patients may show various mutations and amino acid replacement sites at high frequency in the HBV S-region, and these mutations may lead to undetectable Hepatitis B surface antigen (HBsAg), HBsAg antigenic changes or secretion inhibition.

**Supplementary Information:**

The online version contains supplementary material available at 10.1186/s12985-024-02366-2.

## Introduction

Hepatitis B virus (HBV) particles are composed of HBV genome, nucleocapsid and envelope proteins, HBV is a partial double-stranded DNA virus (3.2Kb), belonging to the hepadnaviridae family, prone to mutations, with four open reading frame (ORF), PreS1/S2/S, PreC/C, P, X, PreS1/S2/S encodes three proteins, namely large, medium, and small envelope proteins; PreC/C ORF is responsible for encoding HBeAg and HBcAg; P ORF encodes HBV DNA polymerase; X ORF is responsible for encoding the X protein (HBx) [1].

After HBV enters hepatocytes, the nucleocapsid is released, and the rcDNA (relaxed circular DNA) of HBV enters the nucleus to form cccDNA, which resides in hepatocytes as micro chromosomes [2]. Current anti-HBV nucleotide analogs (NA) have no direct effect on cccDNA, explaining why HBV infection is currently manageable but still incurable with current treatments [1, 3].

Hepatitis B surface antigen (HBsAg) is present on the surface of 22 nm sub viral particles (SVPs) and 42 nm virions. SVPs are spherical or filamentous, composed of protein empty shells. HBV secretes 10^3^ to 10^6^ times more SVPs than virions, so HBsAg is equivalent to SVPs. SVPs are synthesized in the rough endoplasmic reticulum (ER) and secreted through the structural secretion pathway of host cells.

HBsAg is a key serologic marker for detecting HBV infection, but there are also patients with HBsAg-negative HBV infection, known as patients with occult HBV infection (OBI). OBI is defined as the presence of replication-competent HBV DNA in the liver—in the presence or absence of HBV DNA in the blood (usually < 200 IU/ml)—of individuals testing negative for HBsAg assessed by currently available assays [4–6].

The incidence of OBI in different populations varies significantly, and some articles report that the prevalence of OBI in Chinese blood donors is 0.094%, but when combined with other infectious diseases, the prevalence of OBI will increase significantly; for example, the detection rate of OBI in patients with HBV and HIV co-infection is 0.63–88.4%, and the prevalence of OBI screening in liver cancer patients is 40–75% [5, 7, 8].

OBI can be transmitted through blood, liver transplantation, mother and baby, when the patient undergoes chemotherapy or uses immunosuppressants, the host’s immune system will be suppressed, which will lead to hepatitis B reactivation (HBVR), progressive liver disease such as liver cirrhosis (LC), hepatocellular carcinoma (HCC), causing significant damage to the patient’s body. Meanwhile, given its special serological status, it may cause missed diagnosis; therefore, the early intervention and treatment of OBI cannot be ignored, which should arouse the attention of clinical doctors [9–14].

OBI originates from various sources, most likely from patients with chronic hepatitis B (CHB) who have undetectable serum HBsAg but still have a small amount of HBV DNA in the liver [15, 16]. The pathogenesis of OBI and its involvement in liver injury and HCC are still controversial and elusive. To better understand OBI, we need to conduct more studies and long-term clinical follow-up to explore the mechanism of its occurrence. Viral mutation and host immunity jointly affect the occurrence of OBI, and HBV S mutation is currently a research hotspot. Many studies have targeted mutations in the major hydrophilic region (MHR) of HBsAg because it contains the ‘a’ determinant that is a core part of Hepatitis B surface antibody (HBsAb) binding.

From a large amount of clinical data, we found that a small proportion of patients with CHB had serum HBsAg conversion after antiviral drug treatment or on their own; however, serum HBV DNA persisted in the serum at a low level. According to the concept of OBI, it can be considered that this type of patient belongs to OBI, and the patients with HBsAg-/HBV DNA + in the present study may have HBV gene mutation under the action of antiviral drugs or by themselves, resulting in changes in the antigenicity of HBsAg or secretion disorders. It may also be possible that host immune and epigenetic mechanisms inhibit HBV replication activity and viral protein expression.

Herein, we expanded the study area to examine the potential effects of protein-specific amino acid replacement in the HBV S region on HBsAg production and excretion at the cellular level, and explore the possible mechanisms.

## Materials and methods

### Amplification and sequencing of the HBV S region

We collected serum from 107 HBsAg-/HBV DNA + patients at Beijing Youan Hospital, Capital Medical University from September 2020 to December 2022. HBV DNA was extracted using TIANamp Virus DNA/RNA Kit (DP315, TIANGEN BIOTECH (BEIJING) Co.,LTD, China), nested PCR for S-region amplification of extracted DNA.

The primers used in the first round of PCR are PSup3 (5’-TCGCAGAAGATCTCAATCTCG-3’, nt2416-2436) and SB1R (5’-AGGTGAAGCGAAGTGCACAC-3’, nt1577-1596), S1/F (5’-CTCGTGTTACAGGCGGGGTTTTC-3’, nt191-214) and S1R (5’-CATCATCCATATAGCTGAAAGCCAAACA-3’, nt721-748). The primers used in the second round of PCR are PSup4 (5’-CATAAGGTGGGAAACTTTAC-3’, nt2466-2485) and SB2R (5’-TTCCGCAGTATGGATCGGCAG-3’, nt1258-1278), S2/F (5’-TTGTTGACAAGAATCCTCACAATACC-3’, nt215-240) and S2/R (5’-GCCCTACGAACCACTGAACAAATGG-3’, nt686-710).

PCR amplicons were assessed by 1% agarose gel electrophoresis (150 V for 20 min), and positive amplicons were purified. Both strands of purified amplification products were sequenced directly using ABI 3730xl DNA Analyzer. HBV was genotyped based on the full sequence of the S gene using an online prediction tool (https://hbv.geno2pheno.org/index.php) Homology evaluations were performed with the GenBank database using BLAST analysis at https://www.ncbi.nlm.nih.gov. The immune escape mutation of the HBV S protein was determined by the Geno-2-pheno-hbv tool (https://hbv.geno2pheno.org/index.php). The study was approved by the Medical Ethics Committee of Capital Medical University, Beijing Youan Hospital (LL-2019-187-K).

### Selection and construction of candidate S mutations

According to the sequencing results, most of the patients had the HBV C genotype, and we selected the mutation site in the C genotype to construct the mutant plasmid. The 1.05-fold HBV genome (T500-HBV, nt1806-3215/1-1977, C genotype) was chemically synthesized and recombined into a vector to construct a recombinant HBV plasmid that could be replicated and expressed normally, and mutant plasmids was constructed by point mutation and seamless cloning, and the wild-type plasmid was supplied by Dexi Chen (Beijing Institute of Hepatology). The wild-type and mutant plasmids were transfected into Huh7 cells simultaneously, and the transfection efficiency was assessed by the ALP secreted by the wild-type plasmid. The supernatants of the cells were collected at 24 h intervals, and the cell were lysed after 120 h. To study the effect of mutant plasmid on HBsAg concentration, we detected the concentrations of HBsAg in the supernatants and the cell lysates.

### Cell transfection

Before the experiment, the cell plate was prepared for transfection after the cell density reached 50-70% under the microscope. A blank control group (negative control), a wild type plasmid (T500-HBV, positive control) control group and 15 mutant plasmid groups were set up with three compound pores in each group. Six 1.5mL EP tubes were prepared in each group, among which three EP tubes were added with 50uL DMEM medium and 1ug plasmid (1ug/uL). The other three EP tubes were added with 50uL DMEM medium and 3uL transfection reagent (X-tremeGENE HP DNA, Roche, Germany) and placed at room temperature for 10 min. The EP tubes added with plasmid and transfection reagent were mixed in pairs and added into 3 plates of 12-well plates (with 400uL 10%FBS + 1% DMEM double antibody). After six hours, the medium in the 12-well plates was sucked out, and 1mL DMEM medium of 10%FBS + 1% DMEM double antibody was added to each well after washing the medium 2–3 times.

### HBsAg content and HBV DNA titer detection

After transfecting blank control, wild type plasmid and mutant plasmid into Huh7 cells, the cell supernatant was collected every 24 h, and the cell lysate was collected after 120 h. Secreted Alkaline Phosphatase Reporter Gene Assay Kit (Luminescence) (600260-300) was used to detect alkaline phosphatase secreted by the plasmid to evaluate transfection efficiency. HBsAg concentration in cell supernatant and cell lysate collected after transfection was detected by Roche Elecsys E601 (Roche, Germany), the detection reagent is the supporting reagent of the instrument, and the results were calibrated according to transfection efficiency to study the effect of mutant plasmid on HBsAg content.

Abbott M2000 was used to quantitatively detect HBV DNA after transfection for 120 h, and the detection reagent was the matching reagent of Abbott M2000.

### Immunofluorescence staining

Huh7 cells were subjected to cell crawling; after 24 h, the supernatant was discarded and fixed with 4% paraformaldehyde for 15 min, the slides were washed with PBS three times (5 min each time), 1% TritonX-100 was permeabilized at room temperature for 20 min. The slides were washed with PBS three times, 5 min each time, 400ul of sealing solution (100% sheep serum + 5% BSA formulated as 1% sheep serum) was closed for 1 h, Next, we incubated it with primary antibody (biotin rabbit polyclonal antibody against hepatitis B virus surface antigen (Ad/Ay), ab68520) with sealing film at 4℃ overnight, dip washed with PBS three times (5 min each time), incubated with secondary antibody (BA1105) with sealing film to avoid light for 1 h, PBS dip-wash slides three times (5 min each time), dropwise addition of DAPI to the slide, and inverted the crawler on the slide dripping with DAPI. Finally, the slides were observed under the fluorescence microscope.

### Statistical analysis

GraphPad Prism 8.0 were utilized for statistical analysis. Image J software v2.3.0 was used to merge immunofluorescence images.

## Results

### Clinical patient baseline data and S-region mutation characteristics

Among the 107 screened patients, 78 were males and 29 were females, with an average age of 44.46 (34 ∼ 52) years, of which 23 had no history of antiviral therapy and 84 had a history of antiviral therapy. Regarding HBV DNA titers, 50 patients < 10 IU/mL, 44 patients 10–50 IU/mL, and 4 patients 50–100 IU/mL, and 9 patients 100–200 IU/mL (Supplementary Material 1).

Among the 68 sequenced samples, 39 cases were C genotype, 28 cases were B genotype, and 1 case was D genotype. In C genotype, the mutation frequency of V159A, E44G, M47T was higher. In B genotype, the common mutations were N40S, L22V, L49H. The mutation sites of genotype D include T45A, V47L, Q101R and other sites (Supplementary Material 2).

### Cell transfection results

**Fig. 1:**
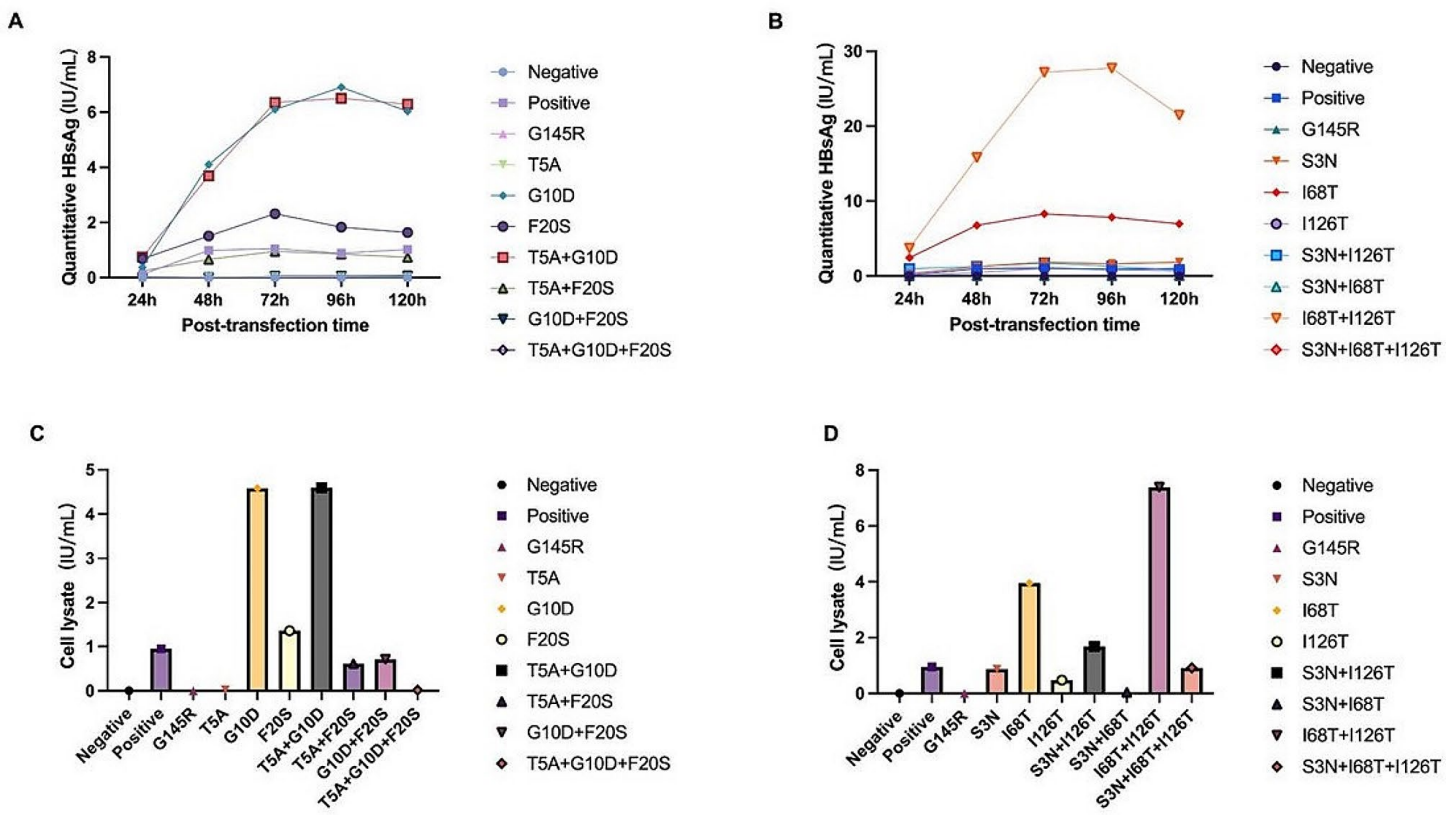
**(A)+(B)** HBsAg content of cell supernatant of mutant plasmid and wild type plasmid in 120 h. **(C)+(D)** HBsAg content in cell lysate after 120h of mutant plasmid and wild type plasmid

Six sites were selected for single or combined mutation, and it was found that single site mutation of sT5A and sI126T could significantly reduce the content of intracellular and extracellular HBsAg. However, single mutation of sS3N could decrease the content of intracellular HBsAg, but the content of cellular supernatant HBsAg would increase. sT5A + sF20S, sG10D + sF20S, sS3N + sI68T combined mutations can significantly reduce the content of intracellular and extracellular HBsAg, and sT5A + sG10D + sF20S and sS3N + sI68T + sI126T combined mutations can also significantly reduce the content of intracellular and extracellular HBsAg. Interestingly, sT5A + sG10D + sF20S has a greater effect on the content of intracellular and extracellular HBsAg, while sS3N + sI68T + sI126T only slightly reduces the expression of intracellular and extracellular HBsAg, and the degree of influence on HBsAg is significantly lower than that of sS3N + sI68T (Table 1 and Fig. 1).

**Table 1:** Effect of S-region mutation on HBsAg

	NC	Positive	G145R	T5A	G10D	F20S	T5A + G10D	T5A + F20S	G10D + F20S	T5A + G10D + F20S	S3N	I68T	I126T	S3N + I126T	S3N + I68T	I68T + I126T	S3N + I68T + I126T
24h	-	0.117	-	-	0.368	0.684	0.741	0.242	-	-	0.311	2.429	0.049	0.953	-	3.7	0.114
48h	-	0.988	-	0.029	4.097	1.508	3.688	0.666	-	-	1.275	6.75	0.455	1.259	0.037	15.819	0.958
72h	-	1.05	0.02	0.034	6.096	2.319	6.348	0.948	0.059	-	1.825	8.286	1.023	1.738	0.069	27.186	1.019
96h	-	0.88	0.019	0.027	6.903	1.833	6.499	0.857	0.059	-	1.641	7.834	0.872	1.279	0.061	27.743	0.854
120h	-	1.02	-	0.026	6.032	1.637	6.288	0.734	0.063	-	1.84	6.961	0.661	0.793	0.051	21.463	0.99
Cell lysate	-	0.947	-	0.021	4.58	1.357	4.595	0.616	0.712	0.013	0.867	3.947	0.475	1.686	0.045	7.393	0.919
HBV DNA (IU/mL)	-	1.22×10^7	3.14×10^7	4.41×10^5	1.14×10^6	1.24×10^5	3.91×10^5	2.14×10^6	2.73×10^6	1.08×10^7	7.14×10^4	4.76×10^6	4.36×10^4	1.55×10^7	3.66×10^6	1.55×10^6	1.94×10^5

-: negative; NC: negative control; Unit: IU/mL

The HBV DNA titer (IU/mL) of the cell supernatant at 120 h after transfection was between 10^5 and 10^7, specific information was shown in Table 1.

### Immunofluorescence staining

**Fig. 2:**
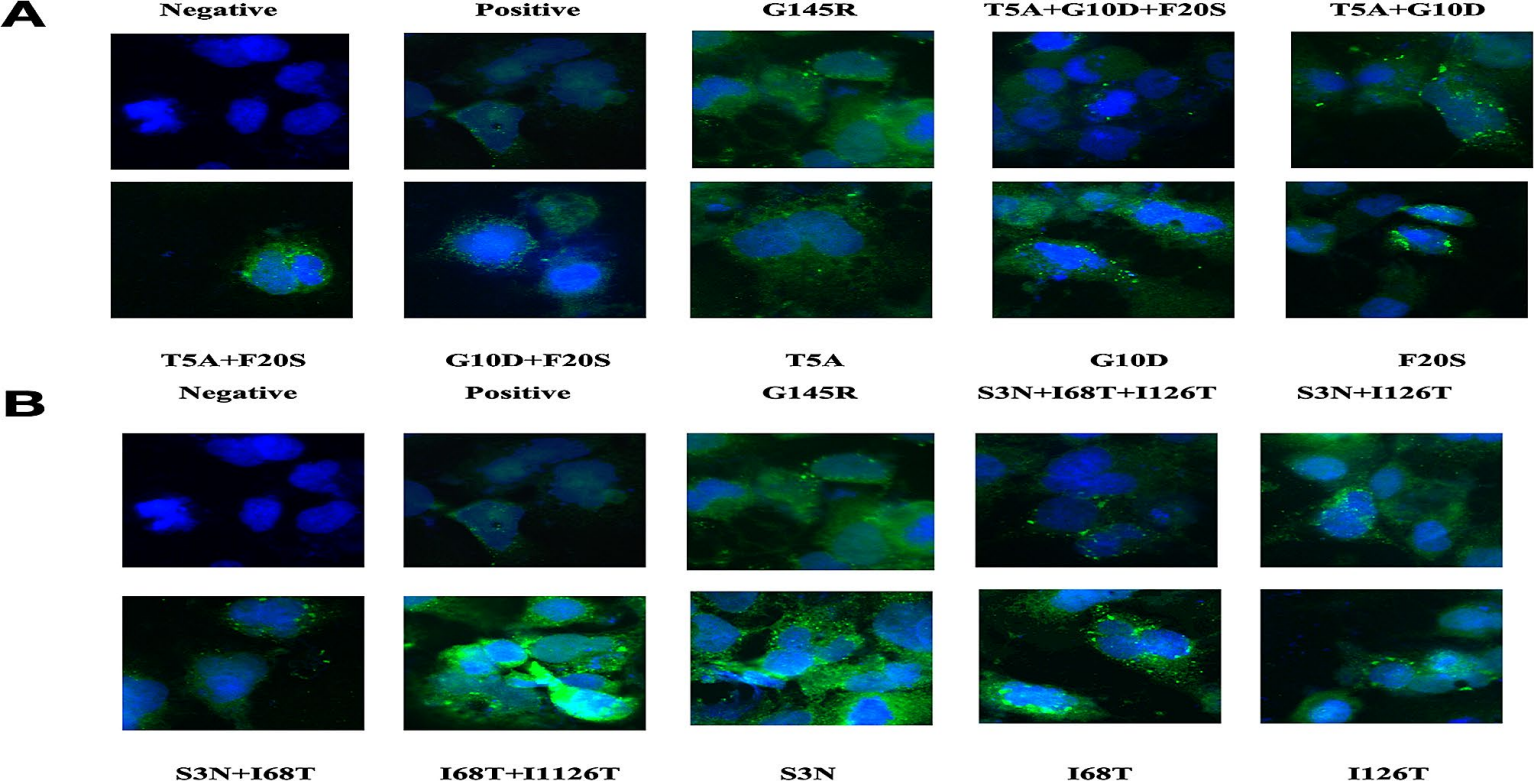
Immunofluorescence staining of HBsAg in Huh-7 cells transfected with wildtype and mutant plasmids. Intracellular HBsAg was stained by FITC (green), and the cell nuclei were stained with DAPI (blue). Magnification, ×400

Wild-type plasmid and mutant plasmids were transfected with cells, and immunofluorescence staining showed that the fluorescence intensity of seven mutant plasmids, sT5A、sT5A + sF20S、sG10D + sF20S、sT5A + sG10D + sF20S、sI126T、sS3N + sI68T、sS3N + sI68T + sI126T were significantly lower than that of the positive control group. This is consistent with our detection results. (Fig. 2).

## Discussion

Currently, the preferred serological marker for diagnosing HBV infection is HBsAg, the earliest serological marker after infection and can be detected 4 to 10 weeks after HBV infection. HBsAg comprises L, M and S surface antigens, and the smallest surface antigen S protein (24 KDa) is 226 amino acids long [1, 17]. HBsAg is important for monitoring the natural infection process of HBV, assessing treatment response and predicting the stage of disease progression. Genotypes B and C are common in Southeast Asia and China [18–20]. Some studies have shown that HBV of genotype C is more infectious and is associated with more serious liver disease, including LC and HCC [21–24].

The 2017 European Association for the Study of Liver Diseases (EASL) clinical guidelines for hepatitis B virus infection divide the natural history of HBV infection into five stages, namely immune tolerance phase, immune clearance phase, immune control phase, reactive phase, and HBsAg negative stage; among them, the fifth stage refers to occult hepatitis B virus infection [16]. It has been reported that the HBV genotype significantly influences the occurrence of OBI [25]. In our study, the mutation rate of the HBV S region was higher in the C genotype than in the B genotype. The MHR of the HBV S gene is between aa100 and aa169, and the ‘α’ determinant is a relatively conserved region in MHR and is the most important immunological determinant in all HBV strains, which is crucial for the detection of HBsAg and the development of HBV vaccine [26]. It has also been proposed in the literature that mutations in the transmembrane structural domain (TMD) gene of HBV S region can also affect the detection of HBsAg [27]. The TMD consists of four parts, including TMD1 (4-24aa), TMD2 (80-98aa), TMD3 (160-193aa), and TMD4 (202-222aa), and it has been examined that mutations in the gene of different regions of the TMD affect the HBsAg expression, which may be because mutations affect the expression of hydrophilic and hydrophobic amino acids.

Patients presenting with HBsAg-negative HBV infection are likely due to a combination of host immune control and viral genomic variation. Mutations, insertions, or deletions in the S gene may affect the antigenicity, immunogenicity, expression, and secretion of HBsAg, failing the HBsAg assay. It may also reduce or even eliminate viral particle replication and/or secretion, negatively affecting HBsAg presentation [28–30]. HBV S region mutation has a significant impact on the antigenicity of HBsAg, which is the basis for the recognition and clearance of HBsAg by the immune system. When the mutation of S region occurs, the antigen structure of HBsAg may change, making it impossible for antibodies to accurately recognize or bind. For example, G145R is a mutation site that has been widely confirmed to be associated with OBI. This mutation changes the antigenic determinant of HBsAg, resulting in changes in the antigenicity of HBsAg, thereby reducing the sensitivity of HBsAg detection and increasing the missed diagnosis rate of OBI [5, 31]. In addition, novel S-region mutations are also being discovered, which also affect the antigenicity of HBsAg and increase the complexity of disease diagnosis.

The mutation in HBV S region may also affect the secretion and expression level of HBsAg, which is an important indicator to evaluate the degree of viral infection and disease progression. The mutation in HBV S region may lead to the decrease of the secretion and expression level of HBsAg, resulting in the decrease of serum HBsAg concentration and increasing the difficulty of detection [5, 32, 33]. This is one of the reasons why OBI patients are difficult to detect with traditional detection methods.

Previous studies on OBI have mostly been limited to blood donors and few studies have been conducted on clinical patients; however, we further investigated the immune escape mutation site in patients with clinical HBsAg-negative HBV infection in the current study. A large number of literature reports that the OBI strain HBsAg of different geographical origin has a high mutation frequency, and some mutations may cause impaired HBsAg secretion or structural changes [34–37]. Among them, the S-region mutation is generally believed to have a greater impact on the production of HBsAg. To test this hypothesis, we investigated the effect of specific amino acid replacement on HBsAg expression in patients with C-genotype HBsAg-negative HBV infection at the cellular level. In this study, we found that the sT5A mutation in TMD1 and sI126T in the MHR ‘a’ determinant significantly reduced HBsAg expression; moreover, we found some very interesting phenomena that mutations in sG10D, sF20S, sS3N, and sI68T at single sites did not significantly reduce HBsAg expression. However, combination of these mutation sites significantly reduced HBsAg expression. We speculate that these genes may control both the secretion and production of HBsAg, and that mutations at a single site are not sufficient to have a strong effect, but the specific mechanism needs further study.

Meanwhile, there are some limitations to this study. For instance, this study is limited to cross-sectional samples, limited to the S segment of the HBV genome, and it is necessary to establish a larger sample size and longer cohort study in the future to monitor the changes in HBV genome in the development of HBsAg-/HBV DNA + patients.

### Electronic supplementary material

Below is the link to the electronic supplementary material.

Supplementary Material 1

Supplementary Material 2

## Data Availability

No datasets were generated or analysed during the current study.

## Abbreviations

OBI: Occult HBV infection
HBV: Hepatitis B virus
TMD: Transmembrane domain
HBsAg: Hepatitis B surface antigen
ORF: open reading frame
HBx: X protein
rcDNA: relaxed circular DNA
HBsAg: Hepatitis B surface antigen
NA: Nucleotide analogs
SVPs: Sub viral particles
ER: Endoplasmic reticulum
HBVR: Hepatitis B reactivation
LC: Liver cirrhosis
HCC: Hepatocellular carcinoma
HBsAb: Hepatitis B surface antibody
